# Alkane hydroxylase gene (*alkB*) phylotype composition and diversity in northern Gulf of Mexico bacterioplankton

**DOI:** 10.3389/fmicb.2013.00370

**Published:** 2013-12-12

**Authors:** Conor B. Smith, Bradley B. Tolar, James T. Hollibaugh, Gary M. King

**Affiliations:** ^1^Department of Biological Sciences, Louisiana State UniversityBaton Rouge, LA, USA; ^2^Department of Marine Studies, University of GeorgiaAthens, GA, USA

**Keywords:** alkane hydroxylases, AlkB, bacterioplankton, diversity, Gulf of Mexico

## Abstract

Natural and anthropogenic activities introduce alkanes into marine systems where they are degraded by alkane hydroxylases expressed by phylogenetically diverse bacteria. Partial sequences for *alkB*, one of the structural genes of alkane hydroxylase, have been used to assess the composition of alkane-degrading communities, and to determine their responses to hydrocarbon inputs. We present here the first spatially extensive analysis of *alkB* in bacterioplankton of the northern Gulf of Mexico (nGoM), a region that experiences numerous hydrocarbon inputs. We have analyzed 401 partial *alkB* gene sequences amplified from genomic extracts collected during March 2010 from 17 water column samples that included surface waters and bathypelagic depths. Previous analyses of 16S rRNA gene sequences for these and related samples have shown that nGoM bacterial community composition and structure stratify strongly with depth, with distinctly different communities above and below 100 m. Although we hypothesized that *alkB* gene sequences would exhibit a similar pattern, PCA analyses of operational protein units (OPU) indicated that community composition did not vary consistently with depth or other major physical-chemical variables. We observed 22 distinct OPUs, one of which was ubiquitous and accounted for 57% of all sequences. This OPU clustered with AlkB sequences from known hydrocarbon oxidizers (e.g., *Alcanivorax* and *Marinobacter*). Some OPUs could not be associated with known alkane degraders, however, and perhaps represent novel hydrocarbon-oxidizing populations or genes. These results indicate that the capacity for alkane hydrolysis occurs widely in the nGoM, but that alkane degrader diversity varies substantially among sites and responds differently than bulk communities to physical-chemical variables.

## Introduction

Alkanes enter marine environments from many natural and anthropogenic sources (Head et al., 2006; Hu et al., 2009). Natural sources include phytoplankton and bacteria (Blumer et al., 1971; Youngblood and Blumer, 1975; Dunahay et al., 1996) and fluxes from hydrocarbon seeps associated with petroleum reservoirs (Hornafius et al., 1999; Seewald, 2003; Sassen et al., 2004). These natural sources undoubtedly account for the capacity of many bacteria to degrade alkanes. However, concerns about petroleum spills, not natural hydrocarbon occurrences, are largely responsible for research on the distribution, diversity and activities of alkane-degrading bacteria (Vomberg and Klinner, 2000; Röling et al., 2002; Sei et al., 2003; van Beilen et al., 2003; Kloos et al., 2006; Teramoto et al., 2009; Berthe-Corti and Nachtkamp, 2010; Hazen et al., 2010; Lu et al., 2012; Rivers et al., 2013).

Several different enzyme systems that vary in substrate chain length and reaction mechanism initiate bacterial *n-alkane* catabolism (van Beilen and Funhoff, 2007). The particulate (or membrane-associated) non-heme iron alkane hydroxylases (alkane 1-monooxygenases) oxidize substrates with chain lengths ≥C_5_-C_16_. These “alkB” hydroxylases are widely distributed among bacteria (Vomberg and Klinner, 2000; van Beilen et al., 2003; Liu and Shao, 2005; Liu et al., 2007; van Beilen and Funhoff, 2007; Wasmund et al., 2009). They are encoded by three genes, *alkB* for the catalytically active alkane hydroxylase, and *alkG* and *alkT* for rubredoxin and rubredoxin reductase, respectively, (Cappelletti et al., 2011). Though they are variable overall, *alkB* gene sequences contain sufficient conservation for the design of broad spectrum PCR primers, which yield amplicons that contain diagnostic histidine motifs (Kloos et al., 2006). *AlkB* sequence conservation has been exploited in a variety of molecular ecological studies to assess the distribution and diversity of alkane degraders in hydrocarbon-contaminated soils and sediments (van Beilen et al., 2003; Harayama et al., 2004; Kloos et al., 2006; van Beilen and Funhoff, 2007). However, surprisingly few studies have explored alkane degraders in marine systems (Wasmund et al., 2009; Wang et al., 2010a).

Wasmund et al. (2009) analyzed *alkB* diversity in genomic extracts obtained from hydrocarbon seep-associated sediments in the Timor Sea. They observed numerous novel sequences, many of which were related to, but distinct from known alkane oxidizers within the γ-Proteobacteria and Actinobacteria. Diversity was greater in sediments from shallower depths (<100 m) than deeper depths (>400 m), and *alkB* gene copy numbers were elevated in sediments nearest hydrocarbon seeps. Guibert et al. (2012) analyzed alkane degraders in intertidal and shallow sub-Antarctic coastal sediments, and like Wasmund et al. (2009) observed novel phylotypes that appeared to represent a temperature-selected community. In addition, they identified *alkB* phylotypes that were proposed as biomarkers for Antarctic alkane degradation. In contrast, Païssé et al. (2011) found no clear relationship between *alkB* expression and hydrocarbon contamination in sediments from coastal Berre lagoon that were chronically polluted by hydrocarbons. However, this study only investigated polluted sediments, so its relevance for unpolluted systems is uncertain.

Thus far, analyses of alkane degraders in the water column have mostly involved culture-based studies supplemented with determinations of isolate *alkB* sequences (e.g., Wang et al., 2010a; Choi and Cho, 2013), although Wang et al. (2010b) also showed that *alkB* gene abundance ranged from 3 × 10^3^ l^−1^ to 3 × 10^5^ l^−1^ in surface waters around Xiamen Island. In addition, Lu et al. (2012) have used gene probes (GeoChip) to show that relative to uncontaminated waters, *alkB* genes were enriched in the hydrocarbon plume of the Macondo well oil spill. Lu et al. (2012) also attributed *alkB* sequences in the plume to various Proteobacteria (e.g., *Bdellovibrio, Roseobacter*, and *Rhodospirillum*), Firmicutes, and Actinobacteria (e.g., *Gordonia* and *Rhodococcus*), including rather enigmatically the obligate mammalian pathogens, *Mycobacterium bovis* and *M. tuberculosis*; representatives of *Alcanivorax* were either undetectable or present in low abundances.

While clearly informative, these studies have not included spatially extensive analyses of *alkB* distribution and diversity, or comparative analyses of patterns for *alkB* and other genetic markers, e.g., 16S rRNA genes. Thus, it is unclear whether alkane-degrading communities as defined by *alkB* are structured similarly to bulk bacterioplankton communities in unpolluted systems, or whether they respond to different variables. To help address this uncertainty, we have analyzed *alkB* gene sequences derived from clone libraries prepared from genomic extracts of bacterioplankton samples distributed across the northern Gulf of Mexico (nGoM) shelf at depths from 2 m to 1700 m. We have previously used a pyrosequencing-based analysis of 16S rRNA genes from the same and additional samples to characterize nGoM bacterioplankton diversity (King et al., 2013). Results from the latter study indicated that composition was stratified by depth, and that known alkane-degrading genera (especially members of the γ-Proteobacteria) occurred throughout the water column. Therefore, we hypothesized that patterns for *alkB* composition and diversity would mirror those for 16S rRNA genes, and for γ-Proteobacteria and Actinobacteria in particular.

## Materials and methods

### Sample collection and alkB analysis

Bacterioplankton DNA was collected during the March 2010 *R/V Cape Hatteras* cruise GC-5 (30° 07′ N, 088° 02′ W to 27° 39′ N, 093° 39′ W) as described in greater detail by King et al. (2013) and Tolar et al. (2013). Genomic DNA was extracted from bacterioplankton collected by filtering about 1 liter of seawater through 0.2 μm filters. DNA extracts were used for *alkB* PCR in reactions containing 12.1 μL of PCR grade water, 2.5 μL 10X High Fidelity PCR buffer (Invitrogen), 0.2 μL 25 mM dNTP mixture, 1 μL 50 mM MgSO_4_, 5 μL 5X bovine serum albumin (Promega), 1.5 μL each of 10 mM stocks of forward and reverse primers, 0.2 μL Platinum Taq DNA Polymerase High Fidelity (Invitrogen), and 1 μL template DNA. The primers used were alkB-1f 5′-AAYACNGCNCAYGARCTNGGNCAYAA and alkB-1r 5′-GCRTGRTGRTCNGARTGNCGYTG (Kloos et al., 2006). The PCR program consisted of an initial denaturation step (94°C, 3 min), followed by 26 cycles of 94°C (1 min), 61°C (1 min), 68°C (45 s), with a final extension of 10 min at 68°C. Amplicons were visualized by electrophoresis on 0.8% agarose gels. Bands of the correct size were excised from the gels, and DNA was extracted from the gel slices with a Zymoclean gel DNA recovery Kit (Zymo Research) according to the manufacturer's instructions. DNA concentrations of the cleaned reactions were determined using a Nanodrop spectrophotometer.

Cloning reactions were carried out using a CloneJET PCR cloning kit according to the manufacturer's instructions. Three (3) μL of ligation mix was added to 25 μL Genlantis SmartCells and incubated on ice for 30 min before heat shock at 42°C for 45 s. Room temperature SOC medium (125 μL) was added and the cells were incubated at 37°C for 1 h while shaking at 225 rpm. Cells were plated on LB plates containing ampicillin, and colonies (at least 30 per sample) were picked for screening after overnight incubation at 37°C. Picked colonies were added to PCR reactions containing 10.5 μL PCR grade water, 12.5 μL Promega GoTaq^®^ Green Master Mix, and 1 μL each of F pJET primer and R pJET primer from 10 μM stocks. The PCR program consisted of an initial denaturation step (95°C, 3 min), followed by 30 cycles of 94°C (30 s), 60°C (30 s), 72°C (90 s), with a final extension of 10 min at 72°C. Amplicons were visualized by electrophoresis on a 1% agarose gel and purified with UltraClean PCR Clean-up kits (MoBio, Folsom, CA) according to the manufacturer's instructions and then sequenced bi-directionally at the LSU Genomics Facility.

### Data analysis

*AlkB* clone sequences and reference sequences downloaded from the FunGene *alkB* database were aligned with ClustalW (Larkin et al., 2007) and adjusted manually as needed. Clone sequences were screened for the presence of two diagnostic histidine-containing motifs (HNXXHH and HSDHH) that contribute to the alkB protein active site. Sequences with both motifs were retained; sequences lacking a motif were retained if they were substantially similar to other sequences, and otherwise at least 150 residues in length. Sequences not meeting these criteria were eliminated from further consideration. After manually adjusting the alignment, a maximum likelihood phylogenetic tree was created using MEGA ver. 5.05 (Tamura et al., 2011) with 100 bootstrap replications. A distance matrix of clone sequences was prepared using Phylip (Felsenstein, 1989) as input for the Mothur platform (Schloss et al., 2009), which grouped the *alkB* sequences into OPUs using a distance cutoff of 0.20 (Wasmund et al., 2009; Guibert et al., 2012). Mothur was used to determine relative abundance of *alkB* OPUs and to compare distributions among sites. Statistical analyses of *alkB* data were carried out using Mothur and R (R Development Core Team., 2008).

### Accession numbers

Sequences have been deposited in GenBank with accession numbers KF163175-KF613575.

## Results and discussion

A total of 508 clone sequences were obtained from 17 samples representing 9 stations and 14 depths with approximately equal numbers of clones from each. However, after curating the sequences the total number of validated *alkB* gene clones was reduced to 401. Rarefaction analysis indicated OPU “discovery” for each of the libraries was saturated or nearly saturated. Samples from two sites, A6-2 m and F6-2 m, were excluded from additional statistical analyses because the numbers of validated clone sequences (8 and 2, respectively) were too few for meaningful comparisons. Although both of these sites harbored *alkB*-containing populations based on results from a prior survey of 16S rRNA genes (King et al., 2013), they appeared to be dominated by taxa that contained divergent genes that were nonetheless amplified with *alkB* primers. The resulting PCR products contained the HSDHH motif, but lacked HNXXH and otherwise differed substantially from all other validated sequences. The function of these genes is unknown, and could not be inferred from BLAST analysis.

Two additional samples from station A6 (20 m and 1700 m), and one from station D5 (2 m), did not yield *alkB* amplicons at all, in spite of repeated efforts. Reasons for amplification failure with these samples are unclear, since a separate study (King et al., 2013) showed that they each yielded 16S rRNA gene amplicons, including sequences assigned to known alkane-degrading genera (e.g., *Alcanivorax*; King et al., 2013) that were also observed in other samples based on both 16S rRNA and *alkB* gene sequences. Although *alkB* concentrations in the three negative samples might be at or below detection by our PCR protocol, the total amounts of DNA used in each of the PCR reactions was similar, and there is no *a priori* reason to expect substantial variation in relative *alkB* concentrations among samples from similar locations or depths. It also seems unlikely that phylotypes not susceptible to amplification dominated the *alkB*-containing communities in the negative samples. Thus, the lack of amplicons for A6-2 m, A6-1700 m and D5-2 m remains enigmatic.

### OPU abundance and classification

Sequences from the 17 positive samples were clustered into 22 OPUs using a distance cutoff of 0.20. Guibert et al. (2012) used a cutoff of 0.20 and reported 30 OPUs from 202 clones pooled from 5 libraries obtained during an analysis of coastal sub-Antarctic sediments. Wasmund et al. (2009) reported even greater richness (53 OPUs) from 246 clones from Timor Sea sediments, also pooled from 5 libraries and based on a cutoff of 0.20.

The lower AlkB OPU richness for nGoM bacterioplankton relative to Timor Sea and sub-Antarctic sediment samples (Wasmund et al., 2009; Guibert et al., 2012) is consistent with patterns observed for bacterioplankton and sediment communities as a whole, since the latter typically support greater richness than the former based on 16S rRNA gene sequence analyses (Kemp and Aller, 2004). The difference between bacterioplankton and sediment AlkB OPU richness might simply reflect differences in the number of cells extracted for analysis, since even small sediment masses can support much larger communities than the water column. In addition (or alternatively), lower habitat diversity in the water column might select for fewer hydrocarbon-oxidizing phylotypes. Whether or not these differences in richness affect responses to hydrocarbon inputs is uncertain.

**Figure 1:**
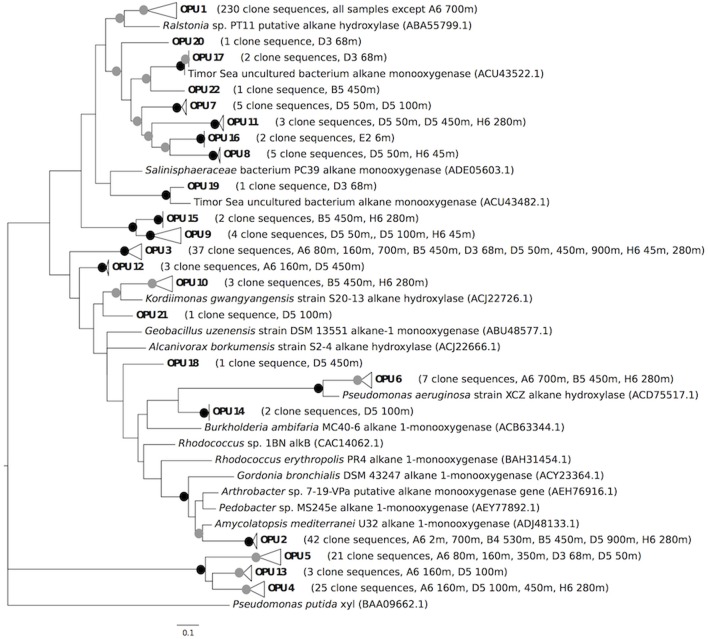
**Maximum likelihood tree comparing putative *alkB* amino acid sequences from this study with reference *alkB* sequences obtained from other studies**. Bootstrap values from 100 resamplings are indicated with black circles for values of 95–100% and gray circles for values of 50–94%. OPUs were determined using a distance cutoff of 0.20 (80% sequence similarity). OPU 1 clusters with the following reference sequences: *Alcanivorax borkumensis* SK2 alkane 1-monooxygenase (CAL18155.1), *Alcanivorax borkumensis* S12-4 alkane hydroxylase (ACJ22702.1), *Alcanivorax dieselolei* S10-17 alkane hydroxylase (ACJ22698.1), *Marinobacter aquaeolei* VT8 alkane 1-monooxygenase (ABM17541.1), and *Marinobacter* sp. S17-4 putative alkane monooxygenase (ACT31523.1). OPU 3 clusters with *Marinobacter adhaerens* HP15 alkane 1-monooxygenase (ADP98338.1), *Marinobacter hydrocarbonoclasticus* S17-4 alkane hydroxylase (ACJ22716.1), and *Marinobacter* sp. P1-14D alkane hydroxylase (ACS91348.1). The tree was rooted with a xylene monooxygenase amino acid sequence from *Pseudomonas putida* (Hara et al., 2004).

Two of the AlkB OPUs identified in this study included reference sequences derived from known alkane degraders. OPU 1, which represented 57.4% of all the AlkB clones (Table 1, Figure 1), included reference sequences from *Alcanivorax borkumensis* SK2, *A. borkumensis* S12-4, *A. dieselolei* S10-17, *Marinobacter aquaeoli* VT8, and *Marinobacter* sp. S17-4. Several of these isolates (*A. borkumensis* S12-4, *A. dieselolei* S10-17, and *Marinobacter* sp. S17-4) were obtained by Wang et al. (2010a) from surface waters of the tropical and sub-tropical southern Atlantic Ocean, while *M. aquaeolei* VT8 was isolated from an offshore oil well in coastal southern Vietnam (Huu et al., 1999). *A. borkumensis* SK2 was isolated from sediments off the northwest coast of Germany (Yakimov et al., 1998).

**Table 1:** **Incidence by sampling site and depth of OPUs detected at 3 or more sampling locations**.

**Site**	**OPU**							
	**01**	**02**	**03**	**04**	**05**	**06**	**09**	**11**
A6-2 m	7	1	0	0	0	0	0	0
A6-80 m	20	0	3	0	1	0	0	0
A6-160 m	18	0	1	3	1	0	0	0
A6-350 m	26	0	0	0	4	0	0	0
A6-700 m	0	23	1	0	0	2	0	0
B4-530 m	28	1	0	0	0	0	0	0
B5-450 m	3	7	6	0	0	1	0	0
D3-68 m	9	0	5	0	7	0	0	0
D5-50 m	11	0	1	0	8	0	2	1
D5-100 m	10	0	0	10	0	0	1	0
D5-450 m	11	0	1	11	0	0	0	1
D5-900 m	24	7	1	0	0	0	0	0
E2-6 m	20	0	0	0	0	0	0	0
F6-2 m	2	0	0	0	0	0	0	0
H6-45 m	10	0	9	0	0	0	1	0
H6-280 m	4	3	9	1	0	4	0	1
MR1-2 m	27	0	0	0	0	0	0	0
Pooled	230	42	37	25	21	7	4	3

OPUs 1 and 3 included reference sequences from Alcanivorax and Marinobacter (see text). OPUs 2, 6, 9 and 11 were associated with Proteobacteria, and OPUs 4 and 5 were unclassified.

OPU 3, which accounted for 9.2% of all clones (Table 1, Figure 1), contained reference sequences from *M*. *adherens* HP15, *M*. *hydrocarbonoclasticus* S17-4, and *Marinobacter* sp. P1-14D. *M. adherens* HP15 was isolated from Wadden Sea diatom aggregates in surface waters (Kaeppel et al., 2012), and *M. hydrocarbonoclasticus* S17-4 was isolated from tropical and sub-tropical southern Atlantic Ocean surface waters (Wang et al., 2010a). Thus, two OPUs that collectively accounted for two-thirds all the nGoM AlkB sequences were closely related to widely distributed *Alcanivorax* and *Marinobacter* isolates.

An additional 85 clone sequences that formed 17 OPUs were distributed among polyphyletic clades comprised of sequences from Actinobacteria, Gammaproteobacteria, and Firmicutes (Figure 1). These 17 OPUs appeared to represent novel phylotypes, some of which were associated with, but distinct from, novel phylotypes from the Timor Sea (Wasmund et al., 2009). However, the lack of congruence between 16S rRNA and *alkB* gene phylogenies (Jurelevicius et al., 2013), and the apparent mobility of *alkB* (van Beilen et al., 2001, 2003, 2005; Smits et al., 2002; Wang et al., 2010b), precluded more specific inferences about OPU affiliations. Irrespective of their phylogeny, however, these unclassified OPUs have not been observed in other AlkB clone libraries (e.g., Wasmund et al., 2009; Guibert et al., 2012). This suggests that the nGoM supports a few widely distributed and dominant alkane degraders along with less abundant, more geographically constrained populations.

A relatively small number of clones (49, 12.2% of the total) forming 3 OPUs (4, 5 and 13) could not be assigned to phyla even tentatively (Figure 1). Sequences of these clones contained the signature AlkB histidine motifs, but their divergence might indicate altered substrate ranges relative to other AlkB proteins, or even different functions. Regardless, these OPUs appear unique to the nGoM.

**Figure 2:**
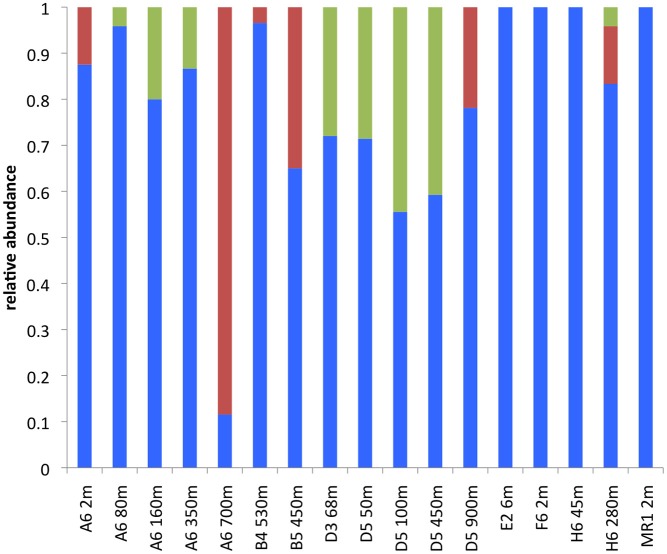
**Phylum level *alkB* composition by sample**. Blue represents Proteobacteria, red represents Actinobacteria, and green represents unclassified.

Of the classified AlkB sequences identified in this study, the most abundant were associated with Gammaproteobacteria, and an *Alcanivorax*-*Marinobacter* OPU in particular (Figures 1, 2; Table 1). Gammaproteobacterial AlkB has also been reported as the dominant phylotype in analyses of Timor Sea sediments and surface waters near Xiamen Island (Wasmund et al., 2009; Wang et al., 2010b). In addition, Hazen et al. (2010) noted that hydrocarbon-oxidizing Gammaproteobacteria, albeit not *Alcanivorax*, dominated microbial communities in the Macondo well oil plume. Thus, Gammaproteobacteria appear to dominate the hydrocarbon oxidizers in many natural marine systems as well as those exposed to chronic or acute hydrocarbon inputs. In contrast, soils appear to harbor more diverse alkane-degrading communities, with a predominance of Actinobacteria (e.g., Jurelevicius et al., 2013).

### OPU distribution

**Figure 3:**
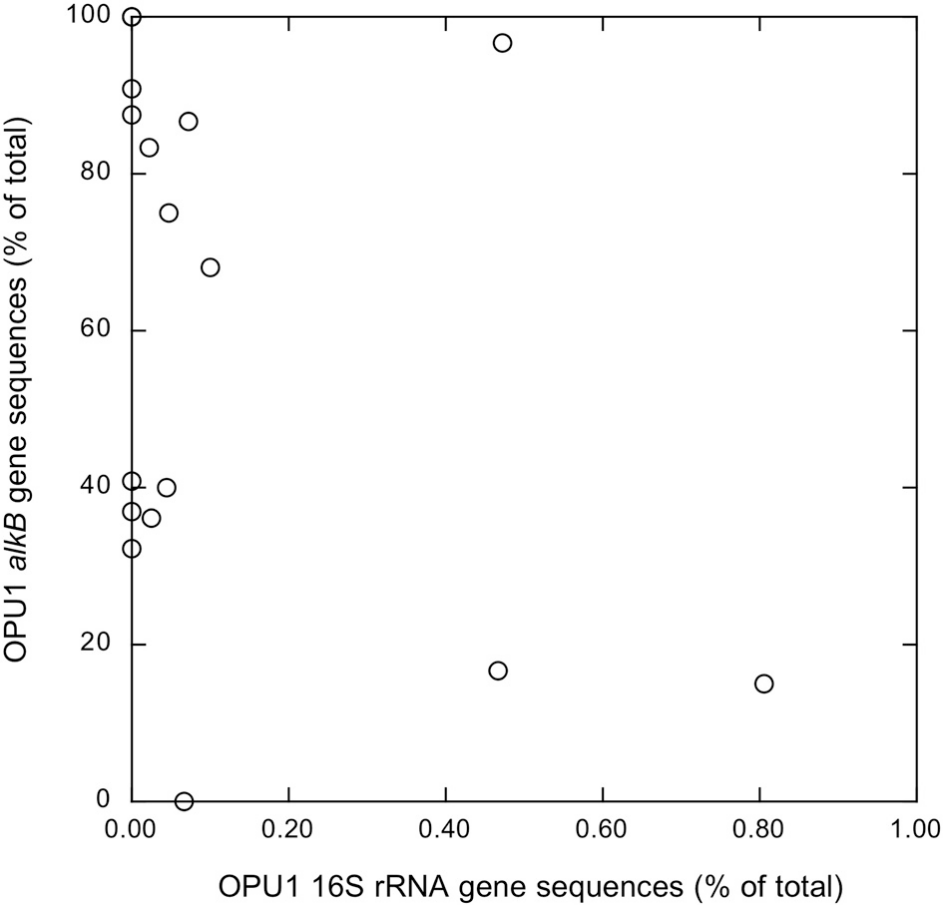
***Alcanivorax*-like *alkB* gene sequence relative abundances of as a function of *Alcanivorax* 16S rRNA gene sequence relative abundances; *alkB* results from this study, 16S rRNA results taken from King et al. (2013)**.

*Alcanivorax*-like AlkB sequences in the dominant OPU 1 were detected in all samples but A6-700 m, even though this sample contained *Alcanivorax* 16S rRNA based on results from a previous analysis (King et al., 2013 and Figure 3). At this site, *Alcanivorax alkB* genes might have been at or near detection limits for our PCR conditions, since 16S rRNA gene sequences attributed to *Alcanivorax* accounted for only 0.07% of the total reads. In contrast, *Alcanivorax*-like *alkB* gene sequences were observed in 7 samples (A6-2 m, D5-50 m, D5-100 m, D5-450 m, E6-2 m, F6-2 m, and MR1-2 m) where *Alcanivorax* 16S rRNA genes were not detected (Figure 3). The lack of concordance between *Alcanivorax* 16S rRNA and *Alcanivorax*-like *alkB* gene sequences at these sites might be explained by the presence of *Alcanivorax*-like *alkB* genes in taxa other than *Alcanivorax* (e.g., *Marinobacter*). More generally, there was no significant correlation between the relative abundance of *Alcanivorax*-like *alkB* sequences and the relative abundance of *Alcanivorax* 16S rRNA gene sequences (Figure 3).

**Figure 4:**
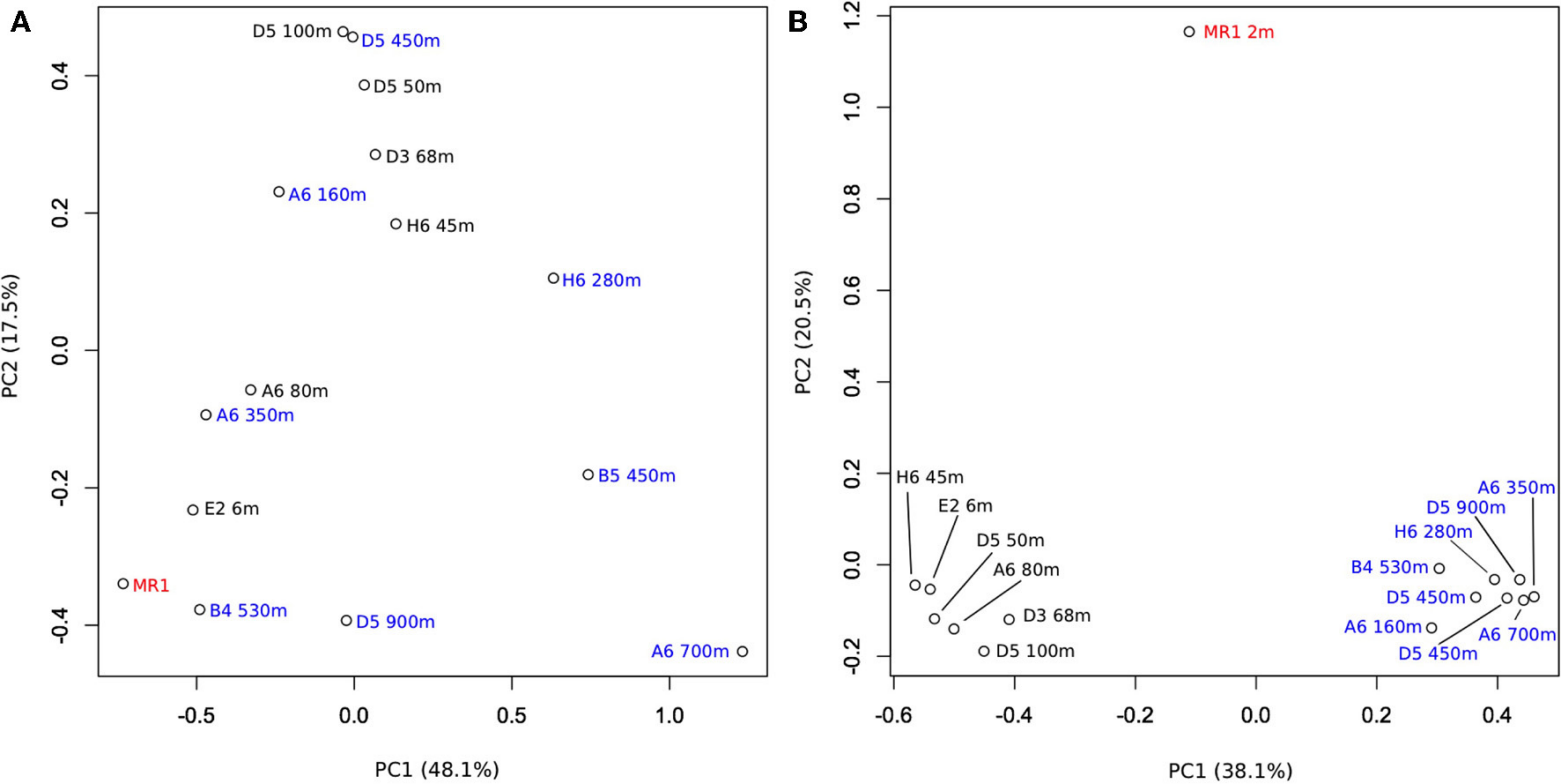
**(A)** Results of a principal component analysis of *alkB* gene OPU composition for 15 nGoM sites designated by location and depth. **(B)** Results of a principal component analysis of 16S rRNA gene OTU composition for the same 15 nGoM sites (data from King et al., 2013). Samples from depths >100 m, ≤100 m and the Mississippi River plume are indicated by blue, black, and red, respectively. Composition data were analyzed after an arcsine transformation.

PCA analysis showed that the composition of AlkB OPU assemblages did not vary consistently with depth or sampling station (Figure 4A), since there were no coherent clusters with separation on axes 1 or 2. These results were consistent with inferences from a similarity plot constructed using the structure-based θ_YC_ calculator (SI Figure 1), which also indicated that AlkB OPUs were not clustered by location or depth. Similarly, results from a canonical correlation analysis (CCorA) revealed no consistent relationships among OPUs and depth (SI Figure 2), or several other physical-chemical variables available for the samples (e.g., salinity, fluorescence, beam attenuation (a measure of particle density), dissolved oxygen, pH and temperature). Removing the most abundant OPUs from the PCA analysis (OPU1 and OPU3) resulted in a cluster of shallow water samples (≤100 m depth) that included one deeper water sample (A6-350 m); the remaining deep-water samples were dispersed across both axes (SI Figure 3). This suggests that the less abundant OPUs in surface waters might form similar assemblages across sites, but that variability in the more abundant OPUs obscures patterns.

Results similar to those for a PCA with all AlkB OPUs were obtained from a PCA analysis of the relative abundances in each sample of 16S rRNA gene sequences for *Alcanivorax, Marinobacter, Pseudomonas, Hydrocarboniphaga,* and *Kordiimonas*, the primary alkane-oxidizing reference taxa with which some of the clone sequences were identified (and SI Figure 4 and King et al., 2013). The distribution of these taxa did not vary consistently with depth. In contrast, PCA analysis of 16S rRNA gene sequences for the bulk bacterioplankton communities in these same samples showed that they separated distinctly by depth (Figure 4B) as previously reported for a larger set of nGoM samples (King et al., 2013; Tolar et al., 2013).

These results collectively indicate that the composition of nGoM alkane-degrading communities varies in response to as yet unidentified biological or abiological factors that do not change consistently with sample depth. This pattern differs from that for bulk bacterioplankton, the composition of which appears to be governed by depth-dependent variables (King et al., 2013; Tolar et al., 2013). In particular, the relative abundance of nGoM Gammaproteobacteria, which constitute the majority of classifiable alkane degraders based on 16S rRNA gene and *alkB* sequences, depends strongly on depth (King et al., 2013).

However, a greater relative abundance of Gammaproteobacteria does not necessarily imply a greater abundance of alkane degraders, since there was limited, albeit significant, positive correlation between the two (*r* = 0.518, *p* = 0.033; SI Figure 5). For example, Gammaproteobacteria at D5-900 m constituted >40% of the total bacterial community (King et al., 2013), yet only 0.1% of this community was identified as alkane degraders. In contrast, samples from A6–160 m, A6–350 m, A6–700 m, B5–450 m, and H6–45 m harbored greater relative abundances of alkane degraders, but lower relative abundances of Gammaproteobacteria (King et al., 2013).

### Indices of AlkB diversity

**Figure 5:**
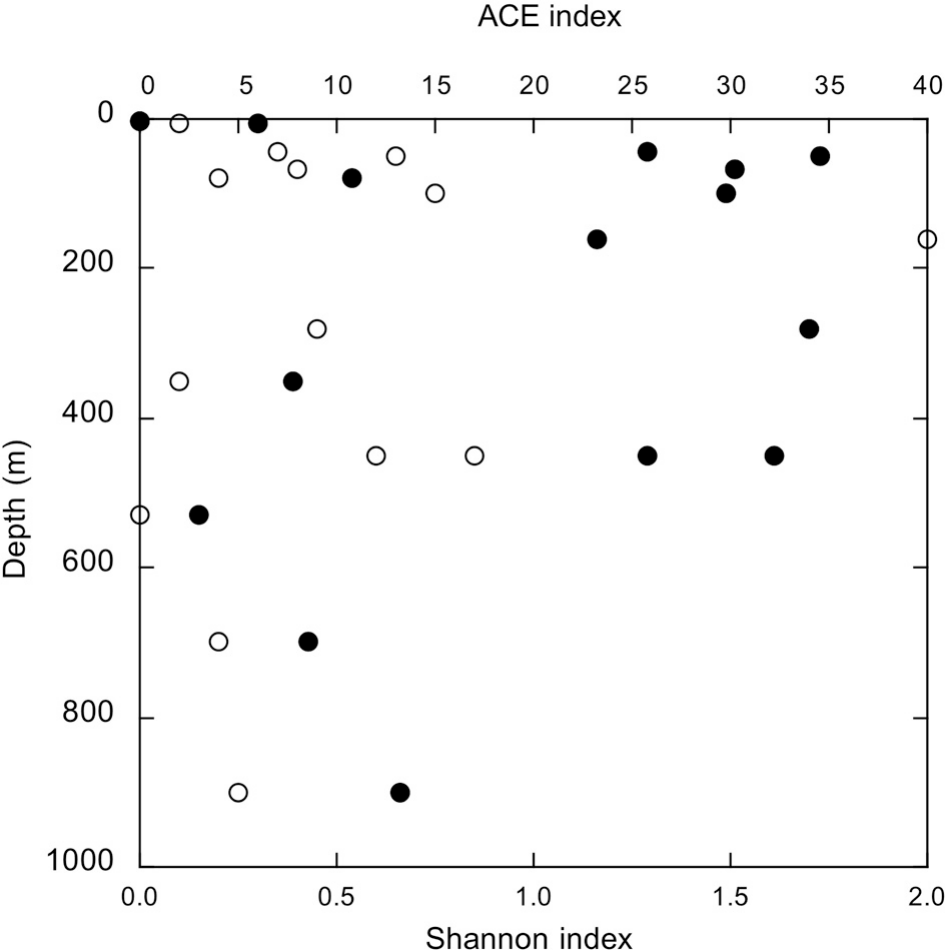
***AlkB* diversity indices as a function of depth; open symbols, ACE index and closed symbols, Shannon index**.

Statistical measures of diversity (e.g., Chao1, ACE, Shannon and Inverse Simpson's indices, and evenness indices) also did not vary consistently with depth or sampling station (Figure 5, SI Table 1). Values for the Shannon index, for example, fell between 0.0 (MR1-2 m, 1 OPU) and 1.79, but there were no systematic differences among samples. Likewise, there were no systematic differences among samples for other diversity indices. In this respect, spatial trends for AlkB diversity were comparable to those for bulk nGoM bacterioplankton based on 16S rRNA gene sequences; diversity metrics for the latter also did not vary with depth or geographic location on the shelf (King et al., 2013).

The absence of spatial patterns in nGoM *alkB* gene sequence diversity suggests that at least during the sampling period (March, 2010), assembly of alkane-degrading communities depended on a common mechanism (e.g., neutral assembly; Emerson and Gillespie, 2008) irrespective of local conditions. A different pattern might emerge, however, during summer stratification and the establishment of hypoxia in surface waters. Changes in substrate availability and quality, temperature and dissolved oxygen could enrich specific members of surface but not deeper communities resulting in more distinct spatial patterns. Data from the Macondo Well oil plume also indicate that the introduction of hydrocarbons from spills or natural sources can create bloom conditions that alter both richness (Hazen et al., 2010; Valentine et al., 2010) and evenness (Lu et al., 2012; Rivers et al., 2013).

## Summary and conclusions

In summary, results from *alkB* gene sequence analyses show that community structure (composition, richness and diversity) of nGoM alkane-degrading bacteria varies among sites independently of depth and location. The absence of vertical structure contrasts with distinct patterns observed for bulk bacterioplankton communities, but is consistent with the distribution of alkane-degrading genera identified by 16S rRNA gene sequences. Two OPUs, one including *Alcanivorax borkumensis*-like AlkB sequences and a second comprised of *Marinobacter*-like AlkB sequences, accounted for nearly two-thirds of all sequences; the former was widely distributed. In contrast to marine sediment AlkB, nGoM bacterioplankton AlkB appeared OPU-depauperate, and only a small percentage of sequences were not identifiable as Proteobacteria or Actinobacteria. Inconsistencies between AlkB distribution and the distribution of 16S rRNA gene sequences attributable to alkane degraders suggest that both markers may be needed to assess the composition and structure of hydrocarbon-oxidizing communities.

### Conflict of interest statement

The authors declare that the research was conducted in the absence of any commercial or financial relationships that could be construed as a potential conflict of interest.

## Supplementary material

SI Figure 1 **Dendrogram of similarities among communities based on the structure-based θ_**YC**_ calculator for *alkB* OPU compositions**.

SI Figure 2 **Canonical correlation analysis of OPU distributions among samples (Y1) and correlations with environmental variables for each sample (Y2)**. Environmental data from King et al. (2013).

SI Figure 3 **Results of a principal component analysis of *alkB* gene OPU composition for 15 nGoM sites designated by location and depth as in Figure 4A**, but after removal of the two most abundant OPUs: 1 and 3.

SI Figure 4 **Results of a principal component analysis of the distribution among sites of alkane-degrading bacterial genera inferred from 16S rRNA sequence analyses**. Relative abundance data for 16S gene sequence OTUs were calculated using a normalized value of 362 sequences per site. Samples from depths >100 m, ≤100 m and the Mississippi River plume are indicated by blue, black, and red, respectively. The alkane-degrading genera included *Alcanivorax, Marinobacter, Pseudomonas, Hydrocar- bonophaga,* and *Kordiimonas*. Composition data were analyzed after an arcsine transformation.

SI Figure 5 **Relative abundance of gammaproteobacterial alkane-degrading genera identified on the basis of 16S rRNA gene sequences for the sites used in this study as a function of the relative abundance of all gammaproteobacteria in the same samples**. Data from King et al. (2013).

SI Table 1 **Diversity statistics calculated with Mothur**. S_obs_, observed richness; C1, Chao1; H', Shannon index; 1/D, inverse Simpson's index; Cov, coverage. Values in parentheses represent 95% lower and upper confidence limits, respectively, for C1, ACE, H', and 1/D. Because each OPU may be present at multiple sites the sum of S_obs_ at all sites is not equal to the pooled value for S_obs_.

## References

[B1] Berthe-Corti L. Nachtkamp M. (2010). Bacterial communities in hydrocarbon-contaminated marine coastal environments, in Handbook of Hydrocarbon and Lipid Microbiology, ed Timmis K.N. (Heidelberg: Springer-Verlag), 2350–2359

[B2] Blumer M. Guillard R. R. L. Chase T. (1971). Hydrocarbons of marine phytoplankton. Mar. Biol. 8, 183–189 10.1007/BF00355214

[B3] Cappelletti M. Fedi S. Frascari D. Ohtake H. Turner R. J. Zannoni D. (2011). Analyses of both the *alkB* gene transcriptional start site and *alkB* promoter-inducing properties of *Rhodococcus* sp. strain BCP1 grown on *n*-alkanes. Appl. Environ. Microbiol. 77, 1619–1627 10.1128/AEM.01987-10 21193665 PMC3067301

[B4a] Choi A. Cho J. C. (2013). *Thalassolituus marinus* sp. nov., a hydrocarbon-utilizing marine bacterium. Int. J. Syst. Evol. Microbiol. 63, 2234–2238 10.1099/ijs.0.046383-0 23148102

[B4] Dunahay T. G. Jarvis E. E. Dais S. S. Roessler P. G. (1996). Manipulation of microalgal lipid production using genetic engineering, in Seventeenth Symposium on Biotechnology for Fuels and Chemicals (New York, NY: Humana Press), 223–231

[B5] Emerson B. C. Gillespie R. G. (2008). Phylogenetic analysis of community assembly and structure over space and time. Trends Ecol. Evol. 23, 619–630 10.1016/j.tree.2008.07.005 18823678

[B6] Felsenstein J. (1989). PHYLIP - (Phylogeny interface package) version 3.2. Cladistics 5, 164–166

[B7] Guibert L. M. Loviso C. L. Marcos M. S. Commendatore M. G. Dionisi H. M. Lozada M. (2012). Alkane biodegradation genes from chronically polluted subantarctic coastal sediments and their shifts in response to oil exposure. Microb. Ecol. 64, 605–616 10.1007/s00248-012-0051-9 22580956

[B8] Hara A. Baik S. Syutsubo K. Misawa N. Smits T. H. M. van Beilen J. B. (2004). Cloning and functional analysis of *alkB* genes in *Alcanivorax borkumensis* SK2. Environ. Microbiol. 6, 191–197 10.1046/j.1462-2920.2003.00550.x 14871203

[B9] Harayama S. Kasai Y. Hara A. (2004). Microbial communities in oil-contaminated seawater. Curr. Opin. Biotechnol. 15, 205–214 10.1016/j.copbio.2004.04.002 15193328

[B10] Hazen T. C. Dubinsky E. A. DeSantis T. Z. Andersen G. L. Piceno Y. M. Singh N. (2010). Deep-sea oil plume enriches indigenous oil-degrading bacteria. Science 330, 204–208 10.1126/science.1195979 20736401

[B11] Head I. M. Jones D. M. Roling W. F. M. (2006). Marine microorganisms make a meal of oil. Nature 4, 173–182 10.1038/nrmicro1348 16489346

[B12] Hornafius J. Quigley D. Luyendyk B. (1999). The world's most spectacular marine hydrocarbon seeps (Coal Oil Point, Santa Barbara Channel, California): quantification of emissions. J. Geophys. Res. Oceans 104, 20703–20711 10.1029/1999JC900148

[B14] Hu L. Guo Z. Feng J. Yang Z. Fang M. (2009). Distributions and sources of bulk OM and aliphatic hydrocarbons in the surface sediments of the Bohai Sea, China. Mar. Chem. 113, 197–211 10.1016/j.marchem.2009.02.001

[B15] Huu N. B. Denner E. B. M. Ha D. T. C. Wanner G. Stan-Lotter H. (1999). *Marinobacter aquaeolei* sp. nov., a halophilic bacterium isolated from a Vietnamese oil-producing well. Int. J. Syst. Evol. Microbiol. 49, 367–375 10.1099/00207713-49-2-367 10319457

[B16] Jurelevicius D. Alvarez V. M. Peixoto R. Rosado A. S. Seldin L. (2013). The use of a combination of *alkB* primers to better characterize the distribution of alkane-degrading bacteria. PLoS ONE 8:e66565 10.1371/journal.pone.0066565 23825163 PMC3688950

[B17] Kaeppel E. Gärdes A. Seebah S. Grossart H.-P. Ullrich M. S. (2012). *Marinobacter adherens* sp. nov., isolated from marine aggregates formed by the diatom *Thalassiosira weissflogii*. Int. J. Syst. Evol. Microbiol. 62, 124–128 10.1099/ijs.0.030189-0 21335492

[B18] Kemp P. F. Aller J. Y. (2004). Bacterial diversity in aquatic and other environments: what 16S rDNA libraries can tell us. FEMS Microbiol. Ecol. 47, 161–177 10.1016/S0168-6496(03)00257-5 19712332

[B19] King G. M. Smith C. B. Tolar B. Hollibaugh J. T. (2013). Analysis of composition and structure of bacterioplankton communities in the northern Gulf of Mexico. Front. Microbiol. 3:438 10.3389/fmicb.2012.00438 23346078 PMC3548560

[B20] Kloos K. Munch J. C. Schloter M. (2006). A new method for the detection of alkane-monooxygenase homologous genes (alkB) in soils based on PCR hybridization. J. Microbiol. Meth. 66, 486–496 10.1016/j.mimet.2006.01.014 16522338

[B21] Larkin M. A. Blackshields G. Brown N. P. Chenna R. McGettigan P. A. McWilliam H. (2007). ClustalW and ClustalX version 2. Bioinformatics 23, 2947–2948 10.1093/bioinformatics/btm404 17846036

[B22] Liu Z. Lozupone C. Hamady M. Bushman F. D. Knight R. (2007). Short pyrosequencing reads suffice for accurate microbial community analysis. Nucleic Acids Res. 35, e120 10.1093/nar/gkm541 17881377 PMC2094085

[B23] Liu C. Shao Z. (2005). *Alcanivorax dieselolei* sp. nov., a novel alkane-degrading bacterium isolated from sea water and deep-sea sediment. Int. J. Syst. Evol. Microbiol. 55, 1181–1186 10.1099/ijs.0.63443-0 15879252

[B24] Lu Z. Deng Y. Van Nostrand J. D. He Z. Voordeckers J. Zhou A. (2012). Microbial gene functions enriched in the Deepwater Horizon deep-sea oil plume. ISME J. 6, 451–460 10.1038/ismej.2011.91 21814288 PMC3260509

[B26a] Païssé S. Duran R. Coulon F. Goñi-Urriza M. (2011). Are alkane hydroxylase genes (*alkB*) relevant to assess petroleum bioremediation processes in chronically polluted coastal sediments? Appl. Microbiol. Biotechnol. 92, 835–844 10.1007/s00253-011-3381-5 21660544

[B26] R Development Core Team (2008). R: A Language and Environment for Statistical Computing. Vienna: R Foundation for Statistical Computing. ISBN 3-900051-07-0. Available online at: http://www.R-project.org

[B27] Rivers A. R. Sharma S. Tringe S. G. Martin J. Joye S. B. Moran M. A. (2013). Transcriptional response of bathypelagic marine bacterioplankton to the Deepwater Horizon oil spill. ISME J. 7, 2315–2329 10.1038/ismej.2013.129 23902988 PMC3834857

[B28] Röling W. F. Milner M. G. Jones D. M. Lee K. Daniel F. Swannell R. J. (2002). Robust hydrocarbon degradation and dynamics of bacterial communities during nutrient-enhanced oil spill bioremediation. Appl. Environ. Microbiol. 68, 5537–5548 10.1128/AEM.68.11.5537-5548.2002 12406747 PMC129918

[B29] Sassen R. Roberts H. H. Carney R. Milkove A. V. DeFreitas D. A. Lanoil B. (2004). Free hydrocarbon gas, gas hydrate, and authigenic minerals in chemosynthetic communities of the northern Gulf of Mexico continental slope: relation to microbial processes. Chem. Geol. 205, 195–217 10.1016/j.chemgeo.2003.12.032

[B30] Schloss P. D. Westcott S. L. Ryabin T. Hall J. R. Hartmann M. Hollister E. B. (2009). Introducing mothur: open-source, platform-independent, community-supported software for describing and comparing microbial communities. Appl. Environ. Microbiol. 75, 7537–41 10.1128/AEM.01541-09 19801464 PMC2786419

[B31] Seewald J. (2003). Organic–inorganic interactions in petroleum-producing sedimentary basins. Nature 426, 327–333 10.1038/nature02132 14628062

[B32] Sei K. Sugimoto Y. Mori K. Maki H. Kohno T. (2003). Monitoring of alkane-degrading bacteria in a sea-water microcosm during crude oil degradation by polymerase chain reaction based on alkane-catabolic genes. Environ. Microbiol. 5, 517–522 10.1046/j.1462-2920.2003.00447.x 12755719

[B33] Smits T. H. M. Balada S. B. Witholt B. van Beilen J. B. (2002). Functional analysis of alkane hydroxylases from Gram-negative and Gram-positive bacteria. J. Bacteriol. 184, 1733–1742 10.1128/JB.184.6.1733-1742.2002 11872725 PMC134907

[B34] Tamura K. Peterson D. Peterson N. Stecher G. Nei M. Kumar S. (2011). MEGA5: molecular evolutionary genetics analysis using maximum likelihood, evolutionary distance, and maximum parsimony methods. Mol. Biol. Evol. 28, 2731–2739 10.1093/molbev/msr121 21546353 PMC3203626

[B35] Teramoto M. Suzuki M. Okazaki F. Hatmanti A. Harayama S. (2009). Oceanobacter-related bacteria are important for the degradation of petroleum aliphatic hydrocarbons in the tropical marine environment. Microbiology 155, 3362–3370 10.1099/mic.0.030411-0 19541999

[B36] Tolar B. King G. M. Hollibaugh J. T. (2013). An analysis of Thaumarchaeota populations from the northern Gulf of Mexico. Front. Microbiol. 4:72 10.3389/fmicb.2013.00072 23577005 PMC3620491

[B37] Valentine D. L. Kessler J. D. Redmond M. C. Mendes S. D. Heintz M. B. Farwell C. (2010). Propane respiration jump-starts microbial response to a deep oil spill. Science 330, 208–211 10.1126/science.1196830 20847236

[B38] van Beilen J. B. Funhoff E. G. (2007). Alkane hydroxylases involved in microbial alkane degradation. Appl. Microbiol. Biotechnol. 74, 13–21 10.1007/s00253-006-0748-0 17216462

[B39] van Beilen J. B. Li Z. Duetz W. A. Smits T. H. M. Witholt B. (2003). Diversity of alkane hydroxylase systems in the environment. Oil Gas Sci. Technol. 58, 427–440 10.2516/ogst:2003026

[B40] van Beilen J. B. Panke S. Lucchini S. Franchini A. G. Röthlisberger M. Witholt B. (2001). Analysis of *Pseudomonas putida* alkane-degradation gene clusters and flanking insertion sequences: evolution and regulation of the *alk* genes. Microbiology 147, 1621–1630 11390693 10.1099/00221287-147-6-1621

[B41] van Beilen J. B. Smiths T. H. M. Roos F. F. Brunner T. Balada S. B. Röthlisberger M. (2005). Identification of an amino acid position that determines the substrate range of integral membrane alkane hydroxylases. J. Bact. 187, 85–91 10.1128/JB.187.1.85-91.2005 15601691 PMC538836

[B42] Vomberg A. Klinner U. (2000). Distribution of alkB genes within n-alkane-degrading bacteria. J. Appl. Microb. 89, 339–348 10.1046/j.1365-2672.2000.01121.x 10971768

[B43] Wang L. Wang W. Lai Q. Shao Z. (2010a). Gene diversity of CYP153A and AlkB alkane hydroxylases in oil-degrading bacteria isolated from the Atlantic Ocean. Environ. Microbiol. 12, 1230–1242 10.1111/j.1462-2920.2010.02165.x 20148932

[B44] Wang W. Wang L. Shao Z. (2010b). Diversity and abundance of oil-degrading bacteria and alkane hydroxylase (*alkB*) genes in the subtropical seawater of Xiamen Island. Microb. Ecol. 60, 429–439 10.1007/s00248-010-9724-4 20683589

[B46] Wasmund K. Burns K. A. Kurtböke I. Bourne D. G. (2009). Novel alkane hydroxylase gene (*alkB*) diversity in sediments associated with hydrocarbon seeps in the Timor Sea, Australia. Appl. Environ. Microbiol. 75, 7391–7398 10.1128/AEM.01370-09 19820158 PMC2786413

[B48] Yakimov M. M. Golyshin P. N. Lang S. Moore E. R. B. Abraham W. Lünsdorf H. (1998). *Alcanivorax borkurnensis* gen. nov., sp. nov., a new, hydrocarbon-degrading and surfactant-producing marine bacterium. Int. J. Syst. Bacteriol. 48, 339–348 10.1099/00207713-48-2-339 9731272

[B49] Youngblood W. W. Blumer M. (1975). Polycyclic aromatic hydrocarbons in the environment: homologous series in soils and recent marine sediments. Geochim. Cosmochim. Acta 39, 1303–1314 10.1016/0016-7037(75)90137-417760164

