# Supplementary material for: Alkane hydroxylase gene (alkB) phylotype composition and diversity in northern Gulf of Mexico bacterioplankton
Source: Front Microbiol. 2013 Dec 12;4:370. doi: 10.3389/fmicb.2013.00370 (PMC3860262; doi:10.3389/fmicb.2013.00370)
Supplement: SI Figure 1 — Dendrogram of similarities among communities based on the structure-based θYC calculator for alkB OPU compositions. [file Presentation1.PDF]

Supplementary information.

SI Table 1. Diversity statistics calculated with Mothur. S_{obs}, observed richness; C1, Chao1; H', Shannon index; 1/D, inverse Simpson's index; Cov, coverage. Values in parentheses represent 95% lower and upper confidence limits, respectively for C1, ACE, H', and 1/D. Because each OPU may be present at multiple sites the sum of S_{obs} at all sites is not equal to the pooled value for S_{obs}.

Site	Depth	S_{obs}	C1	ACE	H'	1/D	Cov
A6	80 m	3	3 (3, 3)	4 (3, 18)	0.54 (0.20, 0.89)	1.43 (1.09,2.07)	0.96
A6	160 m	6	12 (7, 43)	27 (11, 99)	1.01 (0.55, 1.46)	1.92 (1.34,3.41)	0.84
A6	350 m	2	2 (2, 2)	2 (2,2)	0.39 (0.16, 0.63)	1.31 (1.07,1.71)	1
A6	700 m	3	3 (3, 3)	4 (3,7)	0.43 (0.09, 0.77)	1.28 (1.02,1.72)	0.96
B4	530 m	2	2 (2, 2)	0 (0,0)	0.15 (0.00, 0.38)	1.07 (0.95,1.24)	0.97
B5	450 m	7	13 (8, 44)	17 (10,41)	1.61 (1.24, 1.98)	4.87 (3.25,9.70)	0.8
D3	68 m	6	7 (6, 14)	8 (6,19)	1.51 (1.22, 1.79)	4.41 (3.25,6.88)	0.92
D5	50 m	7	9 (7, 22)	14 (9, 35)	1.55 (1.23, 1.86)	4.20 (2.99, 7.04)	0.89
D5	100 m	7	8 (7, 18)	15 (9, 32)	1.49 (1.14, 1.83)	3.82 (2.74, 6.28)	0.89
D5	450 m	6	8 (6, 21)	12 (7, 49)	1.29 (0.95, 1.63)	3.16 (2.38, 4.71)	0.89
D5	900 m	3	3 (3, 3)	5 (3, 18)	0.66 (0.40, 0.91)	1.67 (1.31, 2.32)	0.97
E2	6 m	2	2 (2, 2)	2 (2, 2)	0.30 (0.02, 0.59)	1.21 (0.98, 1.58)	1
H6	45 m	5	6 (5, 19)	7 (5, 20)	1.29 (1.01, 1.56)	3.45 (2.63, 5.02)	0.92
H6	280 m	7	8 (7, 15)	9 (7, 19)	1.70 (1.39, 2.00)	5.31 (3.49, 11.11)	0.92
MR1	2 m	1	1 (1, 1)	0	0.00	1.00	1
Pooled		22	24 (22, 36)	25 (23, 37)	1.66 (1.51, 1.80)	2.82 (2.48, 3.28)	0.99

SI Figure 1. Dendrogram of similarities among communities based on the structure-based θ_{YC} calculator for *alkB* OPU compositions.

SI Figure 2. Canonical correlation analysis of OPU distributions among samples (Y1) and correlations with environmental variables for each sample (Y2). Environmental data from King et al., 2013.

SI Figure 3. Results of a principal component analysis of *alkB* gene OPU composition for 15 nGoM sites designated by location and depth as in Figure 4.A, but after removal of the two most abundant OPUs: 1 and 3.

SI Figure 4. Results of a principal component analysis of the distribution among sites of alkane-degrading bacterial genera inferred from 16S rRNA sequence analyses. Relative abundance data for 16S gene sequence OTUs were calculated using a normalized value of 362 sequences per site. Samples from depths > 100 m, ≤ 100 m and the Mississippi River plume are indicated by blue, black and red, respectively. The alkane-degrading genera included *Alcanivorax*, *Marinobacter*, *Pseudomonas*, *Hydrocarbonophaga,* and *Kordiimonas*. Composition data were analyzed after an arcsine transformation.

SI Figure 5. Relative abundance of gammaproteobacterial alkane-degrading genera identified on the basis of 16S rRNA gene sequences for the sites used in this study as

a function of the relative abundance of all gammaproteobacteria in the same samples. Data from King et al. (2013).

SI Figure 1.

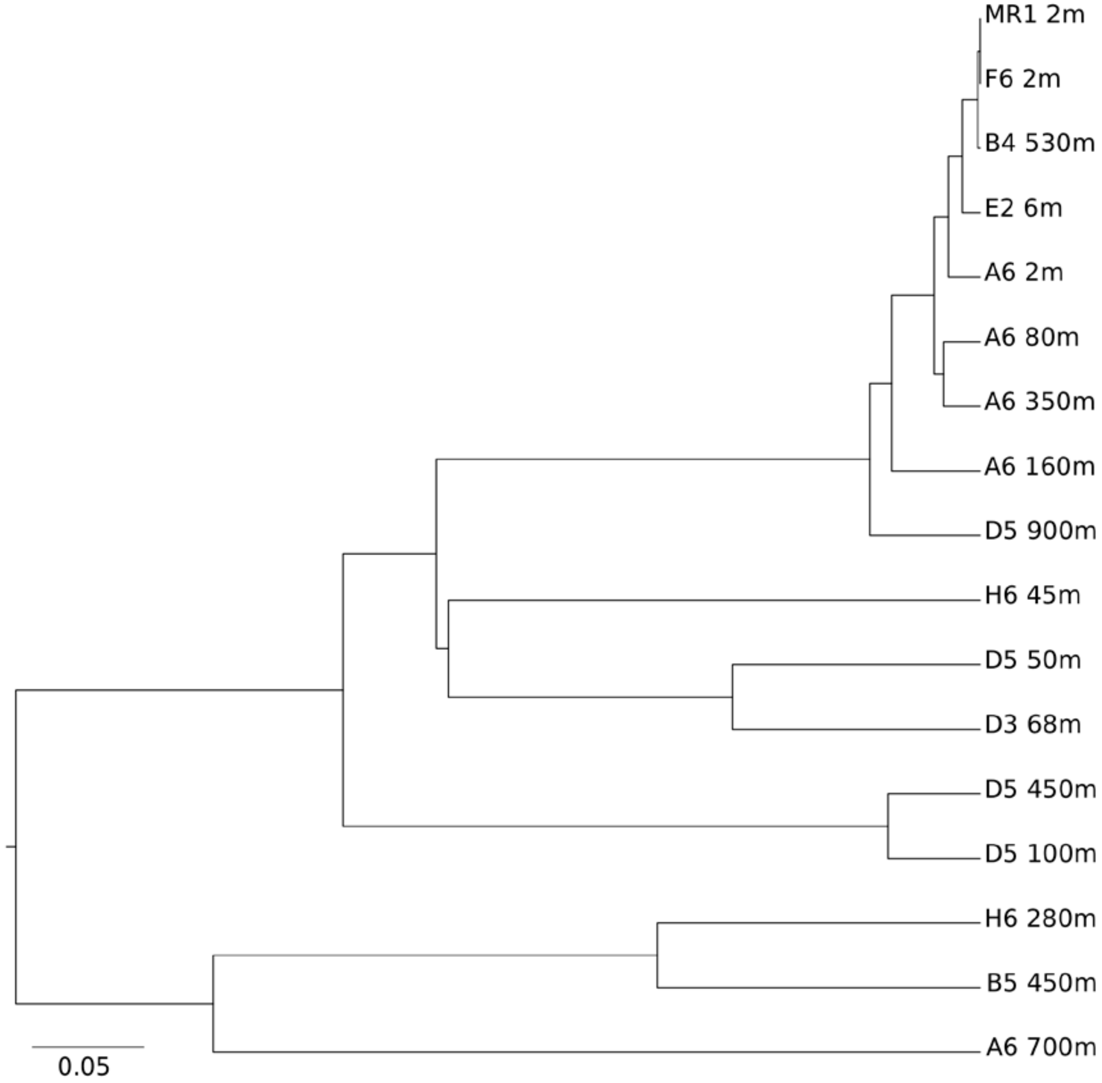

SI Figure 2.

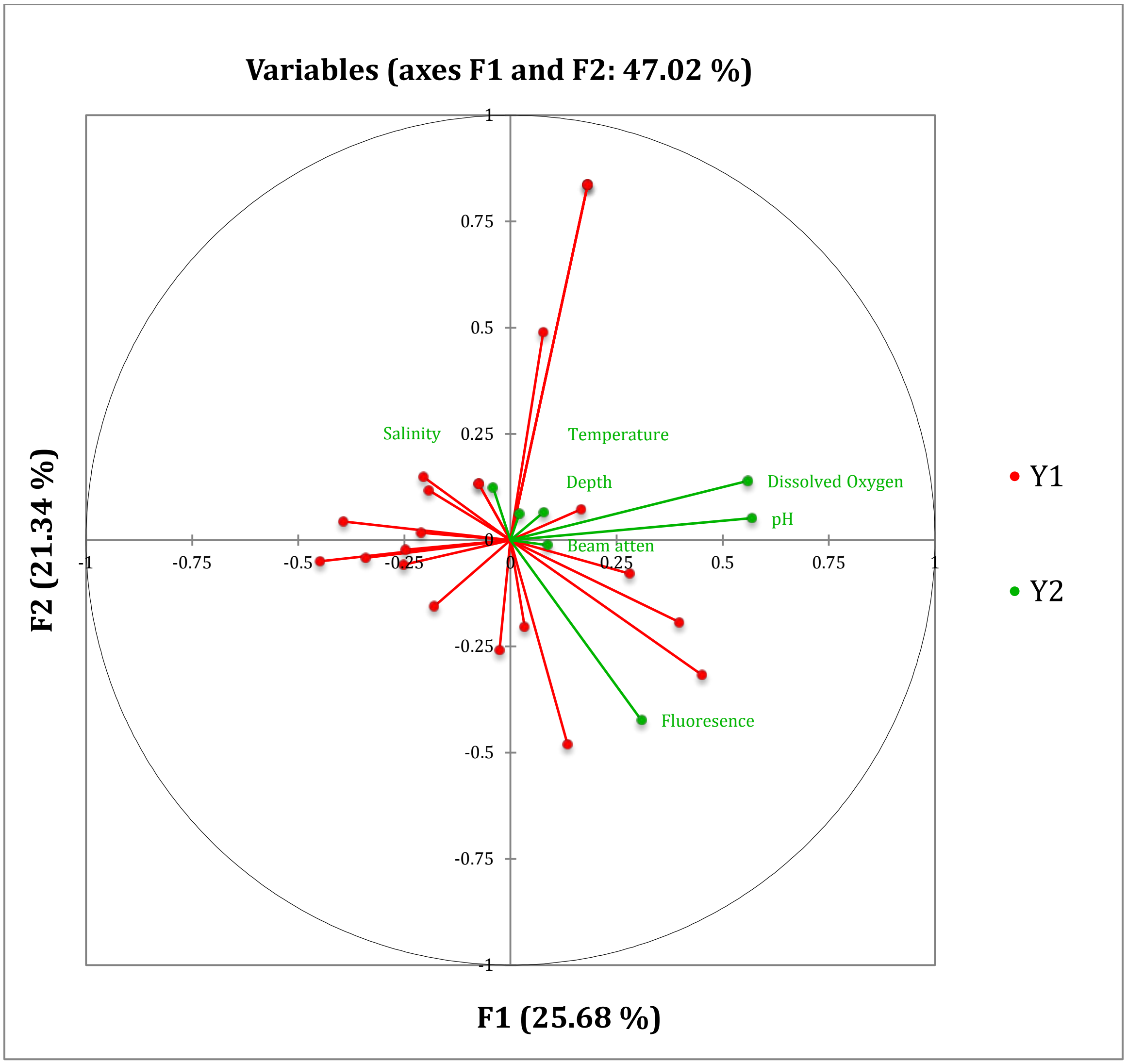
Variables (axes F1 and F2: 47.02 %)
F1 (25.68 %)
F2 (21.34 %)
Salinity
Temperature
Depth
Dissolved Oxygen
pH
Beam atten
Fluoresence
Y1
Y2
-1
-0.75
-0.5
-0.25
0
0.25
0.5
0.75
1

SI Figure 3.

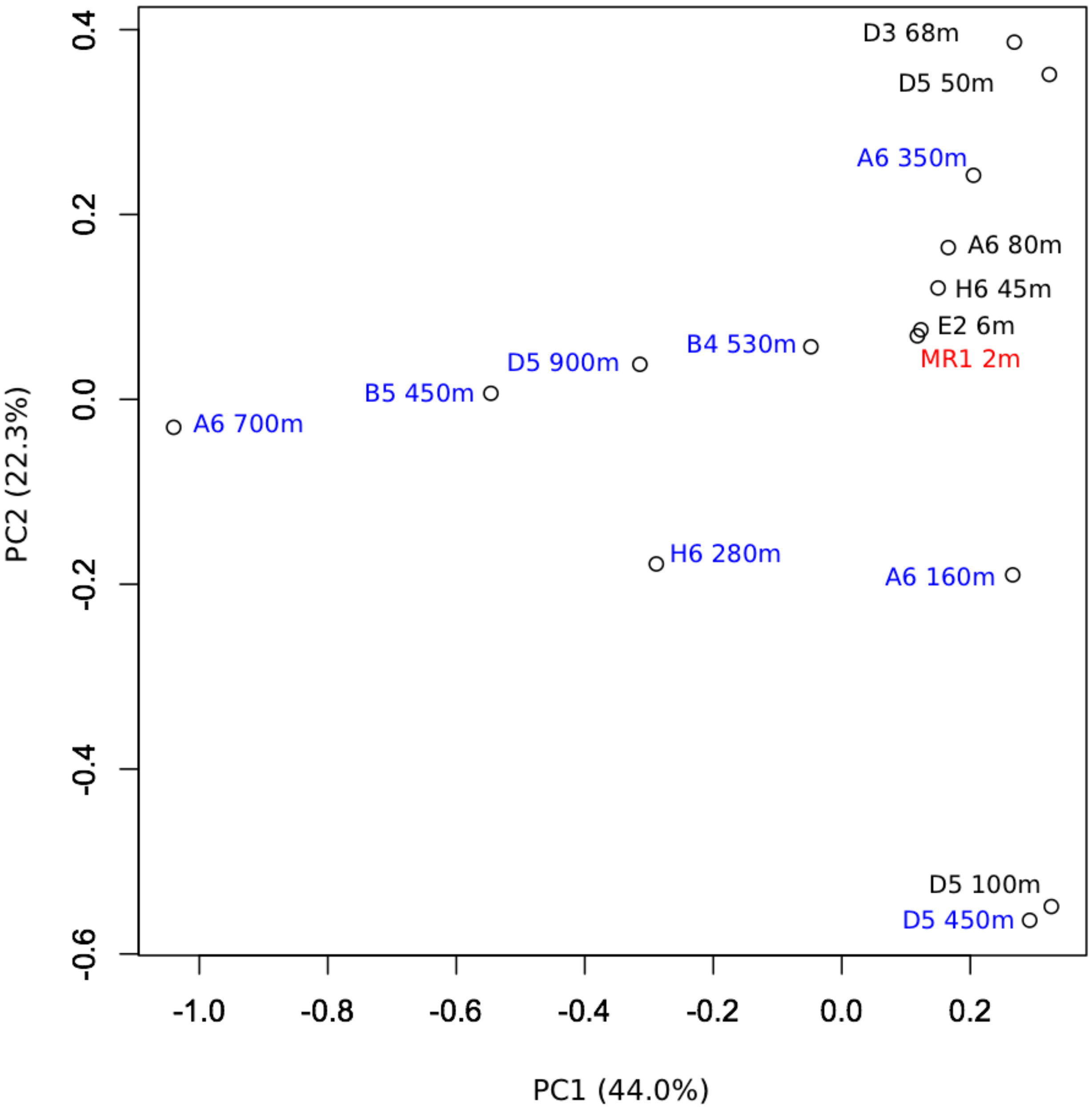
D3 68m
D5 50m
A6 350m
A6 80m
H6 45m
E2 6m
MR1 2m
B4 530m
D5 900m
B5 450m
A6 700m
H6 280m
A6 160m
D5 100m
D5 450m
0.4
0.2
0.0
-0.2
-0.4
-0.6
PC2 (22.3%)
-1.0
-0.8
-0.6
-0.4
-0.2
0.0
0.2
PC1 (44.0%)

SI Figure 4.

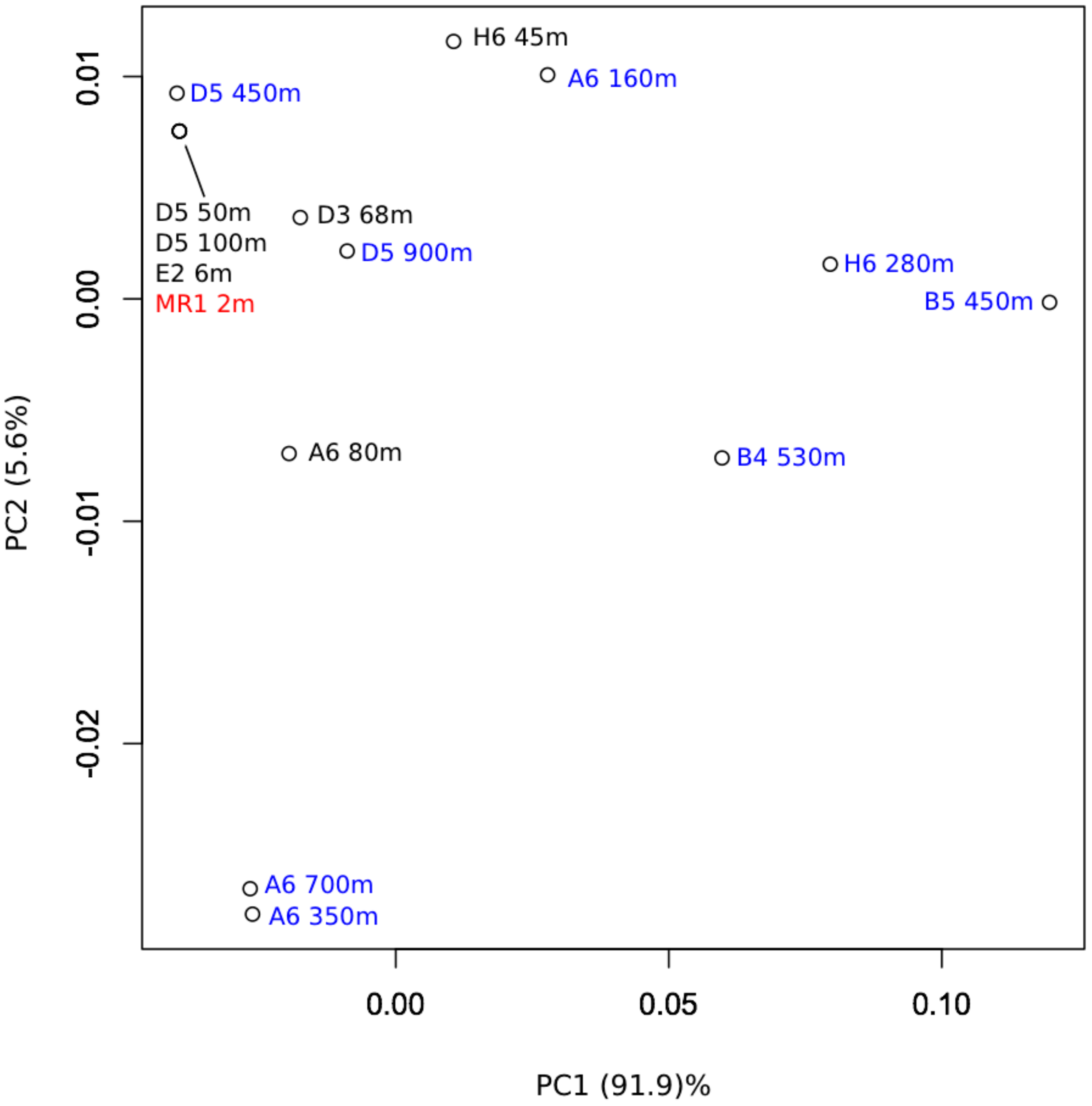
H6 45m
A6 160m
D5 450m
D5 50m
D5 100m
E2 6m
MR1 2m
D3 68m
D5 900m
H6 280m
B5 450m
A6 80m
B4 530m
A6 700m
A6 350m
0.01
0.00
-0.01
-0.02
0.00
0.05
0.10
PC2 (5.6%)
PC1 (91.9)%

SI Figure 5.

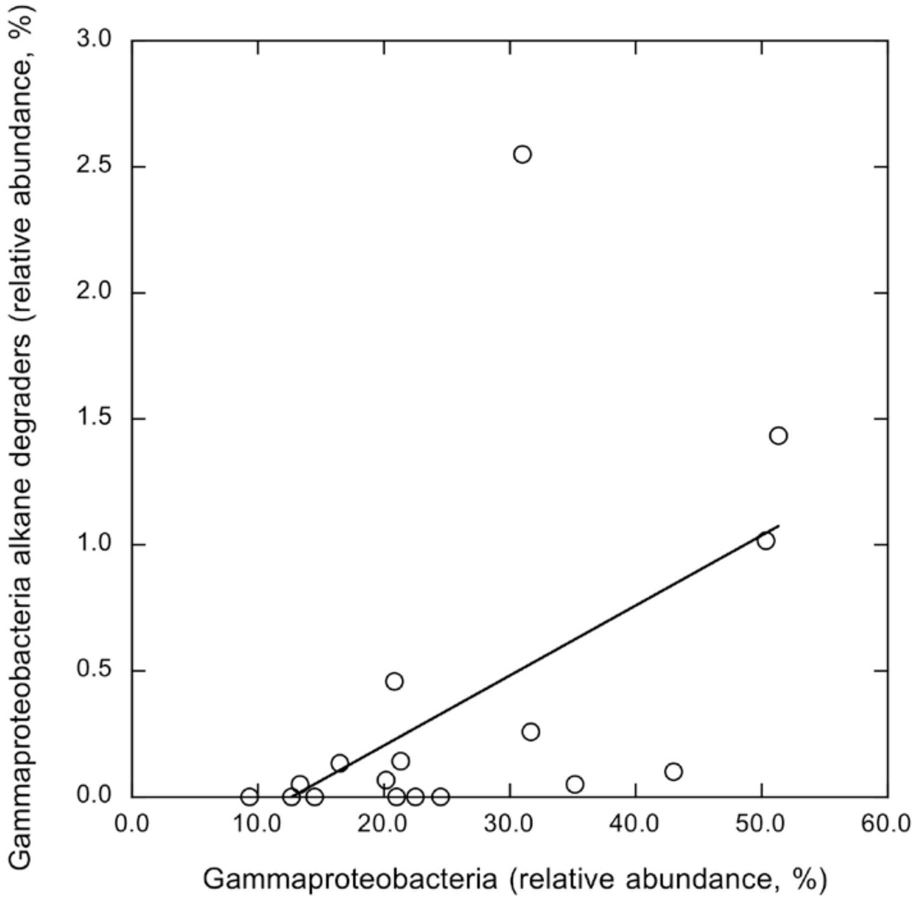
Gammaproteobacteria alkane degraders (relative abundance, %)
3.0
2.5
2.0
1.5
1.0
0.5
0.0
0.0
10.0
20.0
30.0
40.0
50.0
60.0
Gammaproteobacteria (relative abundance, %)
